# Newly Proposed Method With Noise‐Reduction and Smoothing for Computational Fluid Dynamics Using Low‐Resolution Medical Images

**DOI:** 10.1002/cnm.70125

**Published:** 2025-12-04

**Authors:** Yoshiki Yanagita, H. N. Abhilash, S. M. Abdul Khader, K. Prakashini, V. R. K. Rao, Ganesh S. Kamath, Raghuvir Pai, Masaaki Tamagawa

**Affiliations:** ^1^ Graduate School of Life Science System and Engineering Kyushu Institute of Technology Fukuoka Japan; ^2^ Department of Mechanical and Manufacturing Engineering Manipal Academy of Higher Education Manipal India; ^3^ Department of Radiology and Diagnosis, Kasturba Hospital Manipal Academy of Higher Education Manipal India; ^4^ Department of Radiology and Imageology Great Eastern Medical School and Hospital Srikakulam India

**Keywords:** geometry smoothing method, low‐resolution medical image, medical checkups, noise filtering, spline interpolation

## Abstract

Recently, researchers have explored the wall shear stress (WSS) obtained from medical images and computational fluid dynamics (CFD) to provide medical support. However, low‐frequency noise caused by the resolution of the medical images increases the surface roughness of the geometry, thereby reducing the calculation accuracy of WSS. To reduce the surface roughness, regular smoothing methods are applied to geometries obtained from low‐resolution medical images; however volume changes are a problem. In this study, we developed a method to obtain geometries with reduced surface roughness and minimal volume changes from medical images used in checkups, which have low resolution. Our approach combines interpolation of coordinate points with selective removal of low‐frequency noise. This method was applied to 12 carotid artery geometries and one cerebral artery geometry obtained from medical images during the medical checkups; the changes in surface roughness, volume, and WSS in the CFD were compared with before and after smoothing. As a result, we found that the surface roughness of the carotid artery geometries after applying the developed method was approximately 27%–32% smaller than the original geometries, with the volume change remaining minimal, approximately a few percent. The WSS in CFD was found to be approximately 4.2% lower than that of the original geometries. These results demonstrate that our approach improves CFD accuracy for carotid and cerebral arteries, making it useful for medical support based on low‐resolution medical images.

## Graphical abstract

This study presents a method combining coordinate interpolation and selective noise filtering to reduce surface roughness in medical image‐based geometries. Applied to carotid and cerebral arteries, it reduced roughness by 27%–32%, with minimal volume change and a 4.2% decrease in WSS, improving CFD accuracy for low‐resolution medical images.

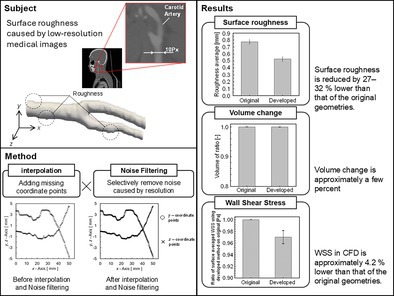

## 1 Introduction

Improving the efficiency and accuracy of medical diagnosis is essential for early disease detection [1]. To realize medical diagnosis using Computational Fluid Dynamics (CFD), various studies have investigated the relationship between vascular diseases and fluid dynamics [2, 3, 4]. However, a major issue is that the calculation accuracy of CFD decreases with the resolution of the medical images [5, 6, 7, 8, 9, 10]. Medical checkup images need a wide imaging range because the location of the disease is unknown, resulting in low resolution (approximately 0.7 mm/pixel). Because the surface roughness of the geometries obtained from these medical images was high, the wall shear stress (WSS) in CFD may be overestimated. Cibis et al. [5, 6] compared WSS in phase‐contrast magnetic resonance angiography (PC‐MRA) and CFD to investigate the effect of the medical image resolution on the computational accuracy of CFD. The WSS in CFD can be obtained by extracting geometry from medical images and solving the equations of motion. PC‐MRA can also be used to calculate the WSS directly from medical images [11, 12, 13]. As a result, the WSS in CFD is higher than that in PC‐MRA. Therefore, medical images for CFD must be at a high resolution to minimize the surface roughness in geometries. However, doctors can perform medical diagnoses using images with resolutions as low as 0.7 mm/pixel. Additionally, obtaining high‐resolution medical images requires longer imaging times and increased radiation exposure, burdening the patient when acquiring images for CFD.

On the other hand, research has been conducted on smoothing methods to reduce the surface roughness of geometries, and Taubin's method is popular [14]. In previous studies using medical images for CFD, Taubin's method has been applied to geometries obtained from high‐resolution medical images to remove high‐frequency noise caused by geometry edges. When applied to geometries obtained from medical images with a low resolution of approximately 0.7 mm/pixel, the calculation error in CFD may become high because the surface roughness cannot be reduced and the volume changes are increased [15]. This is because the geometries obtained from low‐resolution medical images include both high‐frequency noise and low‐frequency noise. Therefore, to ensure the calculation accuracy of CFD using medical images, it is necessary to develop a process that selectively removes low‐frequency noise owing to its resolution, to reduce surface roughness and volume changes.

In this study, we developed a smoothing method to generate geometries with reduced surface roughness and minimal volume changes from medical checkup images with a wide imaging range and low resolution. Our approach uniquely combined two techniques: (1) spline interpolation to densify the coordinate data extracted from low‐resolution images, and (2) a filtering process based on standard deviation to remove spatially varying noise. This two‐step approach allowed for the selective reduction of both high‐ and low‐frequency noise. The developed method was evaluated based on carotid artery geometries for surface roughness, volume changes, and WSS. In addition, by applying this method to cerebral artery geometries and verifying its effectiveness, we assessed the feasibility of using low‐resolution medical checkup images for CFD, characterized by large samples and wide imaging ranges. We proposed a new method to overcome the resolution limitations that were an issue with conventional CFD using medical images.

## 2 Objective Medical Image and Acquisition Method for Geometry

**FIGURE 1:**
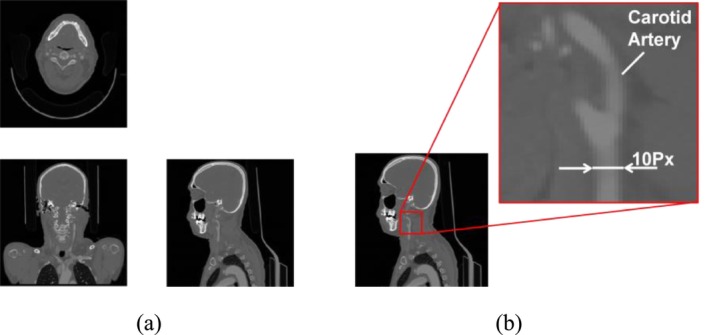
Medical checkup images. (a) Each view and (b) Enlarged view of carotid artery.

**FIGURE 2:**
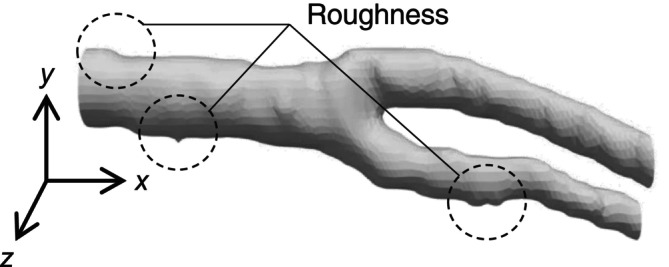
Carotid artery geometry extracted from medical images.

The medical images targeted in this study were 2D‐CT obtained during medical checkups at the Medical Affiliated Hospital of Manipal Academy of Higher Education, India. The imaging ranged from the upper body of the head to the chest, and the resolution of the medical image was 512 × 512 × 394~993 px (cross‐sectional direction × cross‐sectional direction × slice direction). Figure 1 shows an objective medical image. Figure 1a shows each viewpoint, and Figure 1b shows an enlarged view on the right side. The geometries of the carotid and cerebral arteries were obtained from these medical images using ITK‐SNAP [16]. Figure 2 shows an example of the obtained carotid artery geometry. Due to the low resolution of the medical images relative to the diameter, the surface of the obtained geometry appeared uneven. In the objective medical checkup images, the resolution using the carotid arteries cross section was approximately 10 × 10 px of the one slice image (512 × 512 px) used, and the diameter per pixel was approximately 0.7 mm/px (Figure 1b). Therefore, the surface roughness of approximately 10% relative to the diameter was generated.

## 3 Issues in Smoothing for Geometry Obtained From Medical Checkup Images

**FIGURE 3:**
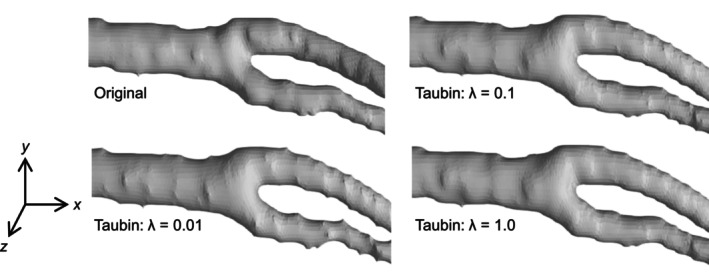
Comparison of original and Taubin‐smoothed geometries from low‐resolution images.

**FIGURE 4:**
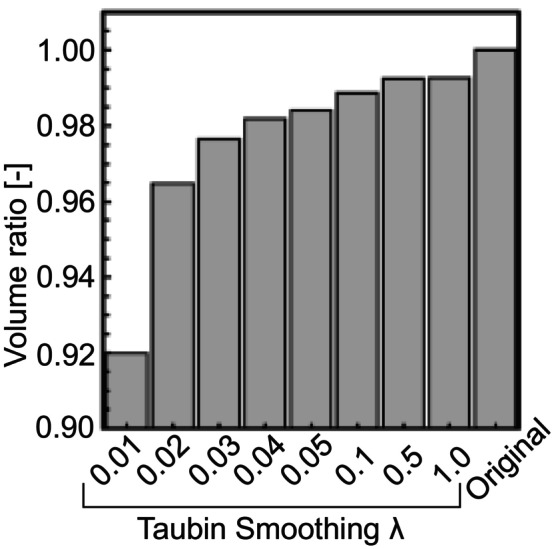
Volume ratio comparison with original and Taubin‐smoothed geometry.

To reduce the surface roughness, a smoothing method is applied to geometries obtained from these medical images. Taubin smoothing methods has been commonly used in the simulation field. When $P_{i}$ is an arbitrary coordinate point to which Taubin's method is applied, and $P_{j}$ is a coordinate point adjacent to $P_{i}$, the amount of correction $∆P_{i}$ by Taubin's method is defined by the following equation:

(1) $$∆P_{i}=\sum_{j\in N\left(P_{i}\right)}w\left(P_{j}-P_{i}\right)$$

where w is the weight coefficient for adjusting $∆P_{i}$. The effect of varying the weighting coefficient on the geometry was examined. Figure 3 shows the original geometry and geometry obtained by Taubin's method. When the weighting coefficient decreased, the surface roughness of approximately 10% relative to the diameter was transformed into a spike (Figure 3). Figure 4 shows the volume changes in both the original geometry and geometry obtained by Taubin's method. When the weighting coefficient decreased, the volume changes increased. Therefore, when Taubin's method is applied to geometries obtained from medical images for checkups, the accuracy of CFD may be reduced by generating a spike and changing the volume. The noise in the geometries obtained from medical images for checkups can be classified into two types: the low‐frequency noise caused by surface roughness of approximately 10% relative to the diameter, and high‐frequency noise above the surface roughness of low‐frequency. Because the amounts of corrections required for high‐and low‐frequency noise differ, this method cannot effectively reduce both types of noise. A band‐pass filter is generally utilized to reduce low‐frequency noise. However, part of the geometry is removed as noise when using a band‐pass filter because its frequency overlaps with the low‐frequency noise components. Therefore, we aim to develop a method for obtaining geometries with low surface roughness and small volume changes by identifying the frequencies of the geometry and low‐frequency noise, and using a process that selectively removes only low‐frequency noise.

## 4 Smoothing Method

### 4.1 Preprocessing1 (Interpolation in Cross‐Sectional Direction)

**FIGURE 5:**
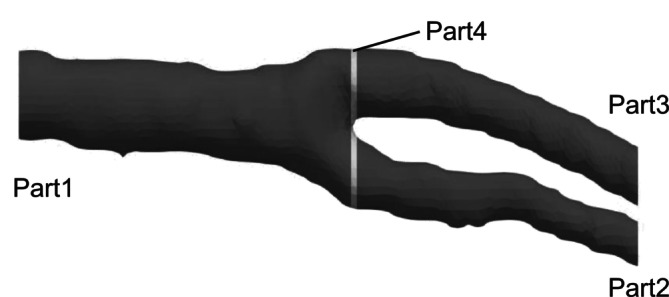
Segmentation of 3D geometry into parts.

**FIGURE 6:**
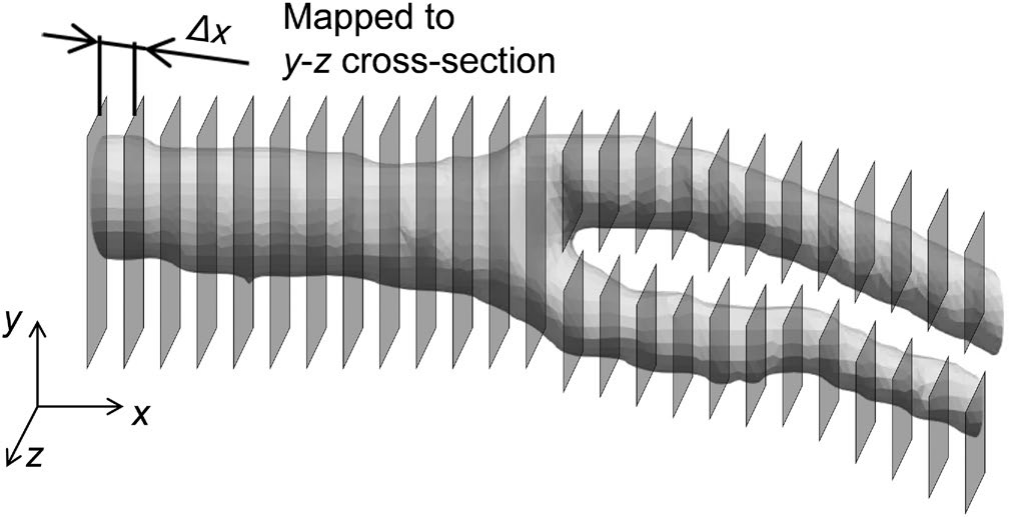
3D geometry segmented by *y‐z* planes.

**FIGURE 7:**
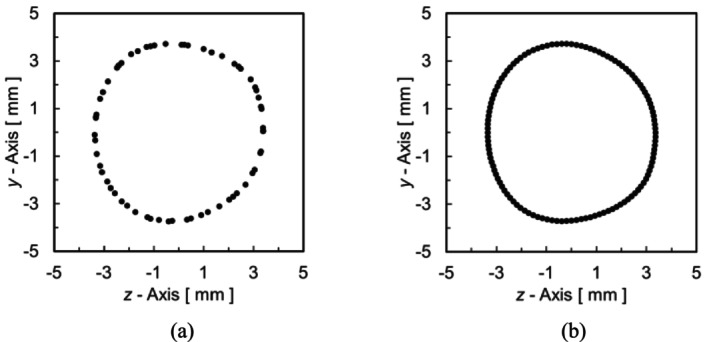
Mapped coordinate points on *y‐z* plane. (a) *Δx* interval coordinate points with mapped on to *y‐z* plane and (b) *y‐z* plane of coordinate points obtained with *Δ*L interval.

To increase the coordinate points of the geometry during preprocessing, interpolations were performed separately in cross‐sectional and slice directions. First, to perform this interpolation, the carotid artery geometry was divided into four parts. A right‐handed Cartesian coordinate system was used for the smoothing method, with the *x*‐axis defined as the slice direction of the medical image (Figure 2). Figure 5 shows the geometry in Figure 2 divided into four parts. Next, a plane was defined based on the divided geometry, and the coordinate points on this plane were linearly interpolated. Figure 6 shows the defined *y*‐*z* plane. The coordinate points on the plane were obtained by mapping the coordinate points within ⊿*x* shown in Figure 6, onto the *y*‐*z* plane. The coordinate points are obtained at equal spaced ⊿L, by linear interpolation. This same process was performed on each plane, as shown in Figure 6. Figure 7 shows the coordinate points on the plane. Figure 7a shows the coordinate points on the *y*‐*z* plane mapped from points within ⊿*x*, while Figure 7b shows the coordinate points after linear interpolation to those from Figure 7a.

**FIGURE 8:**
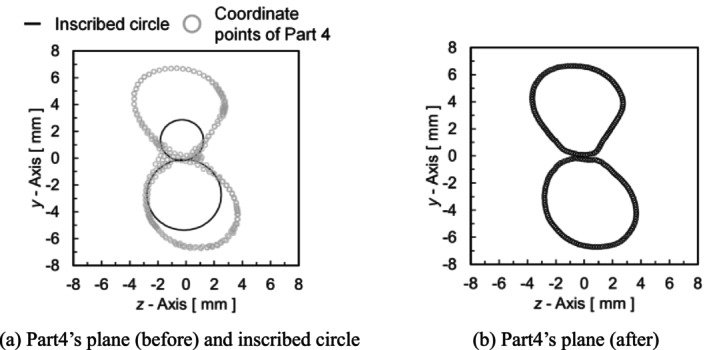
*y*‐*z* plane in Part 4.

Figure 8 shows the plane in Part4. Figure 8a shows the inscribed circles and the plane in Part4, while Figure 8b shows the plane in Part4, excluding the coordinate point before bifurcation. Since the coordinate points in Part4 mix with those before and after bifurcation, as shown in Figure 8a, they need to be classified. First, the inscribed circles are created using the coordinate points from Part2 and Part3. There are three reference points for creating the inscribed circles: the bifurcation point, and one point each from Part2, and Part3. The bifurcation point is the coordinate point in Part4 located on the line segment generated from the two points where the coordinate points on each plane in Part2 and Part3 are most adjacent to each other. Subsequently, the coordinate point in Part4 on the inscribed circles, created from the three reference points, was set as a new plane in Part4, and linear interpolation was performed based on this coordinate point. Using these processes, the coordinate points before and after the bifurcation were classified (Figure 8b).

### 4.2 Preprocessing 2 (Interpolation in Slice Direction)

**FIGURE 9:**
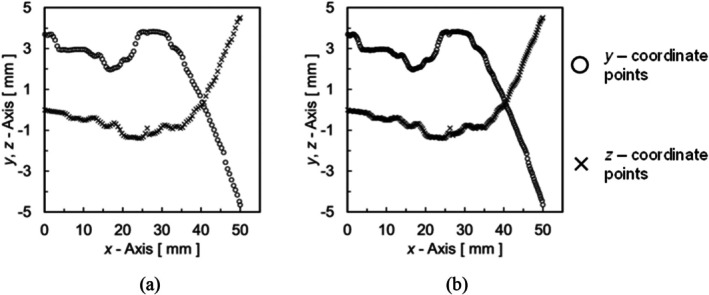
Spline interpolation results (0.1 mm intervals). (a) Reference point and (b) After spline interpolation.

The number of coordinate points in the slice direction is increased by spline interpolation using the reference points from the cross‐sectional direction. The spline functions for the *y* and *z* coordinates, shown in the following equations, are obtained by spline interpolation for each coordinate point in each interval [*x*_i_, *x*_i + 1_].

(2) $$y_{i}\left(x\right)=a_{y,i}{\left(x-x_{i}\right)}^{3}+b_{y,i}{\left(x-x_{i}\right)}^{2}+c_{y,i}\left(x-x_{i}\right)+d_{y,i}$$

(3) $$z_{i}\left(x\right)=a_{z,i}{\left(x-x_{i}\right)}^{3}+b_{z,i}{\left(x-x_{i}\right)}^{2}+c_{z,i}\left(x-x_{i}\right)+d_{z,i}$$

where $a_{y,i}$~$d_{y,i}$ and $a_{z,i}$~$d_{z,i}$ are the Spline coefficients for each interval, and $x_{i}$ represents the *x*‐coordinate of the reference point. The boundary condition of the Spline function in each interval ensures that the coordinate position, and its first and second partial differential values, are the same at the endpoints of each Spline function. Figure 9 shows the *y* and *z* coordinate points before and after Spline interpolation. Figure 9a shows the reference points for Spline interpolation, while Figure 9b shows the coordinate points obtained by Spline interpolation based on Figure 9a.

### 4.3 Noise‐Reducing Process for Low‐Frequency Noise

**FIGURE 10:**
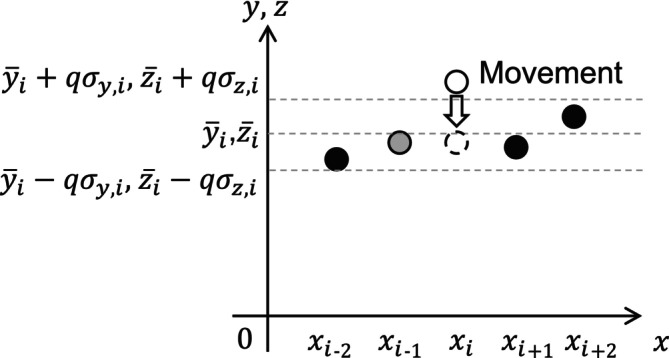
Noise processing based on standard deviation.

**FIGURE 11:**
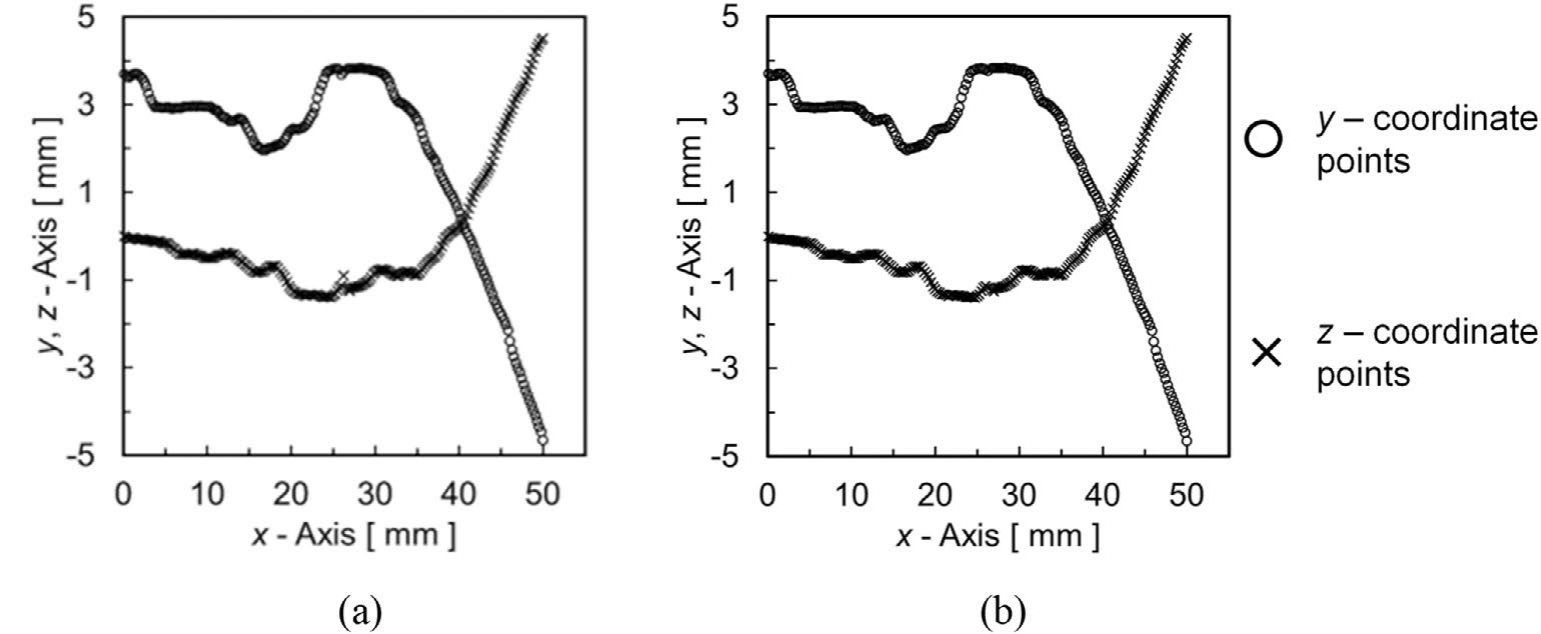
Noise processing applied to Spline curve (0.1 mm interval). (a) Before noise processing (Figure 9b) and (b) After noise processing.

This section explains the noise processing method for reducing low‐frequency noise in medical checkup images, as mentioned in Section 3. Figure 10 shows a schematic of the noise‐reducing process, and the judgment formula is defined by the following equation:

(4) $$\left\{\begin{array}{c} y_{i}=y_{i}\;\text{when}\;\bar{y_{i}}-q\sigma_{y,i}<y_{i}<\bar{y_{i}}+q\sigma_{y,i}\\ y_{i}=y_{i,\mathrm{med}}\;\text{when}\;{\bar{y}}_{i}-q\sigma_{y,i}\geq y_{i},\bar{y_{i}}+q\sigma_{y,i}\leq y_{i} \end{array}\right.$$

(5) $$\left\{\begin{array}{c} z_{i}=z_{i}\;\text{when}\;\bar{z_{i}}-q\sigma_{z,i}<z_{i}<\bar{z_{i}}+q\sigma_{z,i}\\ z_{i}=z_{i,\mathrm{med}}\;\text{when}\;\bar{z_{i}}-q\sigma_{z,i}\geq z_{i},\bar{z_{i}}+q\sigma_{z,i}\leq z_{i} \end{array}\right.$$

where $y_{i,\mathrm{med}}$ and $z_{i,\mathrm{med}}$ are median values, ${\bar{y}}_{i}$ and ${\bar{z}}_{i}$ are average values, $\sigma_{y,i}$ and $\sigma_{z,i}$ are standard deviations, and ${\mathrm{q\sigma}}_{y,i}$ and ${\mathrm{q\sigma}}_{z,i}$ are noise judgment ranges. This process allows for the calculation of the median, average values and standard deviations for the two coordinate points before and after, while any coordinate points outside the range of the average value ± standard deviation are replaced with the median value. Since this process selectively reduces low‐frequency noise while maintaining most coordinate points unchanged, the volume change is considered minimal. Figure 11 shows the coordinate points before and after noise processing. Figure 11a,b shows the coordinate points before and after noise processing. Note that Figure 11a is the same as Figure 9b. Low‐frequency noise can be classified and reduced based on the standard deviation from the noise processing (Figure 10b). By applying this noise processing repeatedly, the low‐frequency noise can be further reduced. In this paper, we evaluate the effectiveness of the method by applying this noise processing once. The coordinate points obtained through these processes were then converted to Standard Triangle Language (STL), resulting in the geometry used for CFD.

## 5 Evaluation Method for Geometry and Numerical Analysis Method

### 5.1 Evaluation Method for Surface Roughness

**FIGURE 12:**
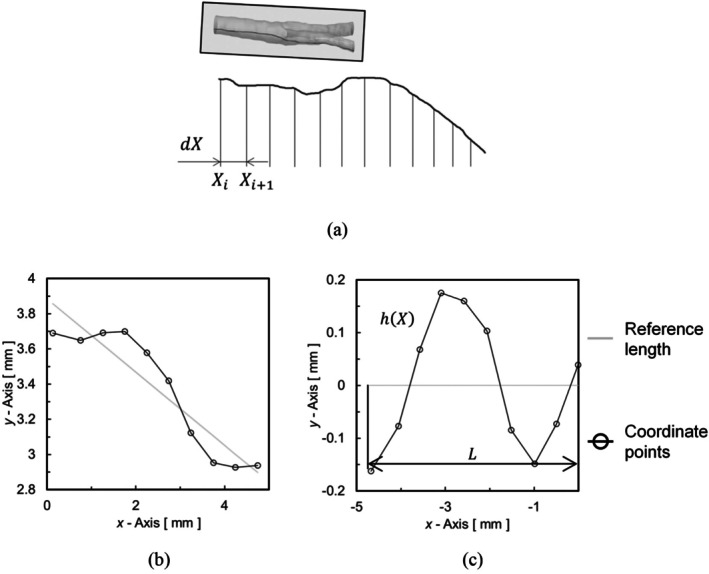
Calculation method for arithmetic mean roughness. (a) Measurement interval of arithmetic mean roughness, (b) Coordinate points and reference length, and (c) After transformed rotational coordinate.

The effectiveness of the developed method was evaluated by surface roughness, volume change, and WSS from CFD. The roughness of the geometry, before and after smoothing, was evaluated using the arithmetic mean roughness. Figure 12 shows an example of the calculation method for arithmetic mean roughness. The coordinate points obtained from the geometry (Figure 12a) were divided into sections at intervals dX = 5 mm, and the reference length in each section was defined as a linear function using the least‐squares method (Figure 12b). These coordinate points and reference lengths were rotated (Figure 12c), and the arithmetic mean roughness ${\mathrm{Ra}}_{i}$ of each section [*X*_i_, *X*_i + 1_] was calculated using the following equation:

(6) $${\mathrm{Ra}}_{i}=\frac{1}{L}\int_{X_{i}}^{X_{i+1}}h\left(X\right)\mathrm{dX}$$

where L is the reference length, and $h\left(X\right)$ is the roughness curve. The averaged‐arithmetic mean roughness $\bar{\mathrm{Ra}}$ was calculated using the following equation:

(7) $$\bar{\mathrm{Ra}}=\frac{1}{N_{\mathrm{Ra}}}\sum_{i=1}^{N_{\mathrm{Ra}}}{\mathrm{Ra}}_{i}$$

where $N_{\mathrm{Ra}}$ is the number of sections. The averaged‐arithmetic mean roughness obtained from each section was used to evaluate the surface roughness.

### 5.2 Evaluation Method for Volume

The volume before and after smoothing was evaluated using the volume ratio of the original geometry and geometry after smoothing. Since only the surface mesh is described in the output STL, volume mesh needs to be created from the surface mesh, and the volume of the geometry is calculated from the volume mesh. Ansys Space Claim is used to create the volume mesh and measure the volume.

### 5.3 Numerical Analysis Method

**FIGURE 13:**
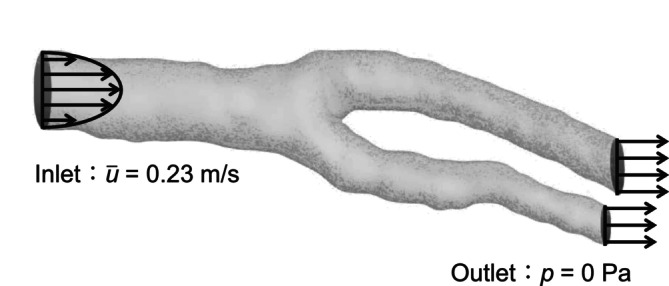
Location of boundary conditions for carotid artery.

**FIGURE 14:**
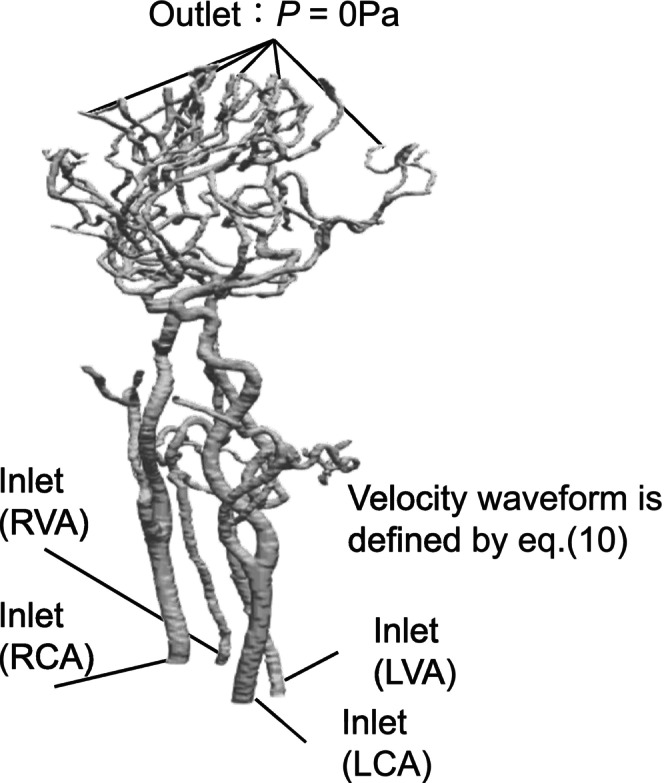
Location of boundary conditions for cerebral artery.

The effects of changes in surface roughness and volume, using the developed method on the WSS in CFD were investigated. The flow field was analyzed using OpenFOAM. The flow is assumed to be incompressible with steady laminar flow (*Re* = 444), and the governing equations are the continuity equation and the Navier–Stokes equation as follows:

(8) ∇·u=0

(9) $$\frac{\partial u}{\partial t}+u\left(\nabla \cdot u\right)=-\frac{1}{\rho }\nabla p+\nu \nabla^{2}u$$

where u is velocity, p is pressure, ρ is density, and ν is dynamics viscosity. Figure 13 shows the locations of boundary conditions for carotid artery, while Table 1 lists these boundary conditions. The inlet boundary condition is defined by the averaged‐velocity ($\bar{u}$ = 0.23 m/s), with a velocity distribution was developed based on the Hagen–Poiseuille equation. The outlet boundary condition was set to atmospheric pressure (0 Pa), and the wall boundary condition is assumed to be a no‐slip. Figure 14 shows the locations of boundary conditions for cerebral artery, and Table 2 lists these boundary conditions. The cerebral artery has four inlet boundary faces: LCA, RCA, LVA, and RVA. For the inlet boundary condition, the velocity was set based on the velocity distribution developed derived from the velocity waveform of the carotid artery. The velocity waveform at these interfaces is defined by the following equation:

(10) $$Q_{\mathrm{art}}\left(t\right)=A_{\mathrm{art}}\beta \left\{a_{0}+\sum_{i=1}^{4}a_{i}\cos\left(\frac{2\mathrm{\pi i}}{T}t\right)+b_{i}\sin\left(\frac{2\mathrm{\pi i}}{T}t\right)\right\}$$

(11) $$u_{\mathrm{art}}\left(r,t\right)=\frac{Q_{\mathrm{art}}\left(t\right)}{A_{\mathrm{art}}}\left(1-{\left(\frac{r}{R}\right)}^{2}\right)$$

where $A_{\mathrm{art}}$ is the cross‐sectional area of each inlet boundary faces, β is the coefficient for adjusting averaged‐velocity of carotid and vertebral arteries, $a_{i}$ and $b_{i}$ are the model coefficients for velocity waveform. Table 3 lists the model coefficients for velocity waveform. The averaged‐velocities in the LCA and RCA were 0.26 m/s. In contrast, the velocity waveforms in the LVA and RVA were defined by adjusting the amplitude of the carotid artery velocity waveform to achieve averaged‐velocity of 0.255 m/s. The outlet boundary condition was set to atmospheric pressure (0 Pa), and the wall boundary condition was assumed to be a no‐slip. The fluid viscosity in the carotid artery and cerebral artery was assumed to be Newtonian, with density ρ = 1050 kg/m^3^ and viscosity μ = 0.004 Pa.s. Additionally, the maximum size of the calculation mesh was set to 0.1 mm, which is smaller than the 0.7 mm unevenness caused by the resolution.

**TABLE 1:** Boundary conditions for carotid artery.

	Velocity [m/s]	Pressure [Pa]
Inlet	$\bar{u}=0.23$	∂p/∂n=0
Outlet	∂u/∂n=0	p=0
Wall	u=0	∂p/∂n=0

**TABLE 2:** Boundary conditions for cerebral artery.

	Velocity [m/s]			Pressure [Pa]
Inlet	LCA RCA	$\bar{u}=0.26$	$Q_{\mathrm{art}}\left(t\right)=$ $A_{\mathrm{art}}\beta \left\{a_{0}+\sum_{i=1}^{4}a_{i}\cos\left(\frac{2\mathrm{\pi i}}{T}t\right)+b_{i}\sin\left(\frac{2\mathrm{\pi i}}{T}t\right)\right\}$	∂p/∂n=0
	LVA RVA	$\bar{u}=0.255$		
Outlet	∂u/∂n=0			p=0
Wall	u=0			∂p/∂n=0

**TABLE 3:** Model coefficients for velocity waveform of Equation (10).

	$A_{\mathrm{art}}$	β	$a_{0}$	$a_{1}$	$a_{2}$	$a_{3}$	$a_{4}$	$b_{1}$	$b_{2}$	$b_{3}$	$b_{4}$
LCA	4.52 × 10^−5^	3.85 × 10^−5^	0.26	−0.10	−0.10	−0.03	−0.014	0.169	−0.002	−0.002	−0.002
RCA	4.80 × 10^−5^										
LVA	1.79 × 10^−5^	3.77 × 10^−5^									
RVA	1.51 × 10^−5^										

## 6 Results and Discussion

### 6.1 Evaluation for Surface Roughness and Volume Changes Before and After Smoothing

**FIGURE 15:**
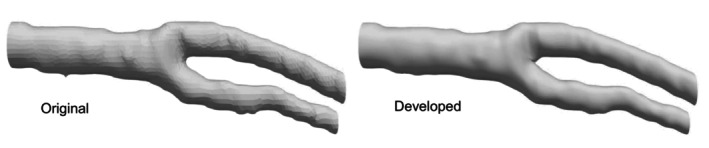
Comparison of geometries before and after smoothing.

**FIGURE 16:**
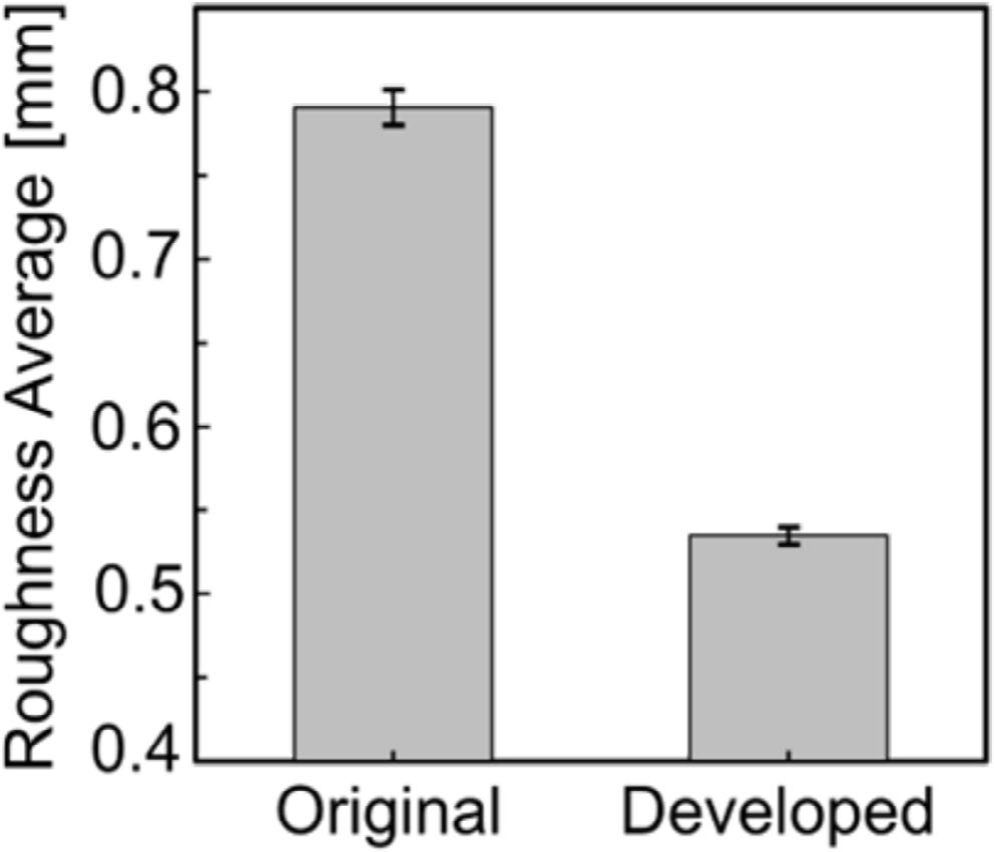
Comparison of averaged‐arithmetic mean roughness before and after smoothing.

**FIGURE 17:**
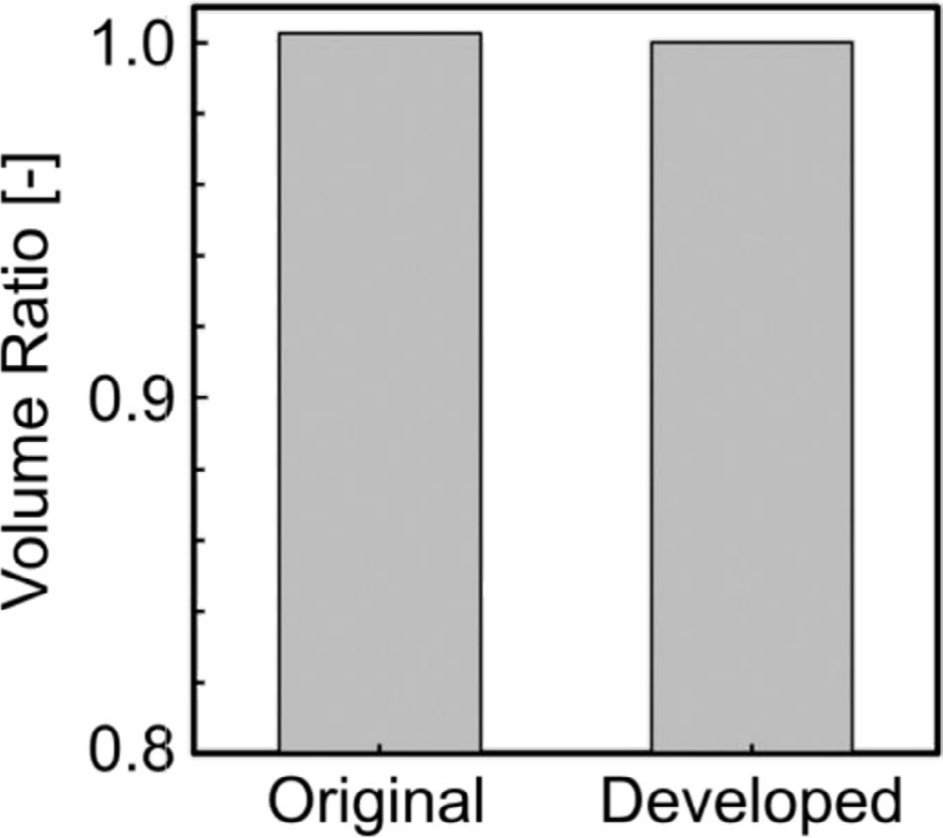
Comparison of volume ratios before and after smoothing.

Figure 15 shows the original geometry and the geometry after the developed method. The carotid artery had a diameter of 7.11 mm and a length of 49.77 mm. The smoothing process took approximately 56 s when executed on a laptop (CPU: Intel Core i5‐7200U, memory: 8 GB). First, the surface roughness before and after smoothing was evaluated using the averaged‐arithmetic mean roughness. Figure 16 shows the surface roughness before and after smoothing. It was found that the averaged‐arithmetic mean roughness of the geometry after smoothing was approximately 31% lower than the original geometry. Next, volume changes before and after smoothing were evaluated using the volume ratio. Figure 17 shows the volume changes before and after smoothing. It was found that the volume change was minimal, with only a few percent difference after smoothing.

### 6.2 Effect of Developed Method on CFD of WSS

**FIGURE 18:**
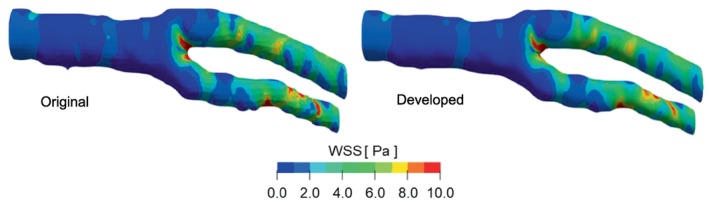
WSS distribution before and after smoothing.

**FIGURE 19:**
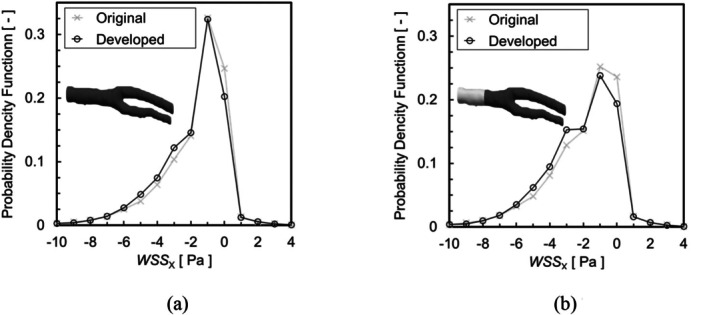
PDF of WSS in the flow direction $\tau_{x}$. (a) All calculation region and (b) Three‐fourths of the calculation region.

**FIGURE 20:**
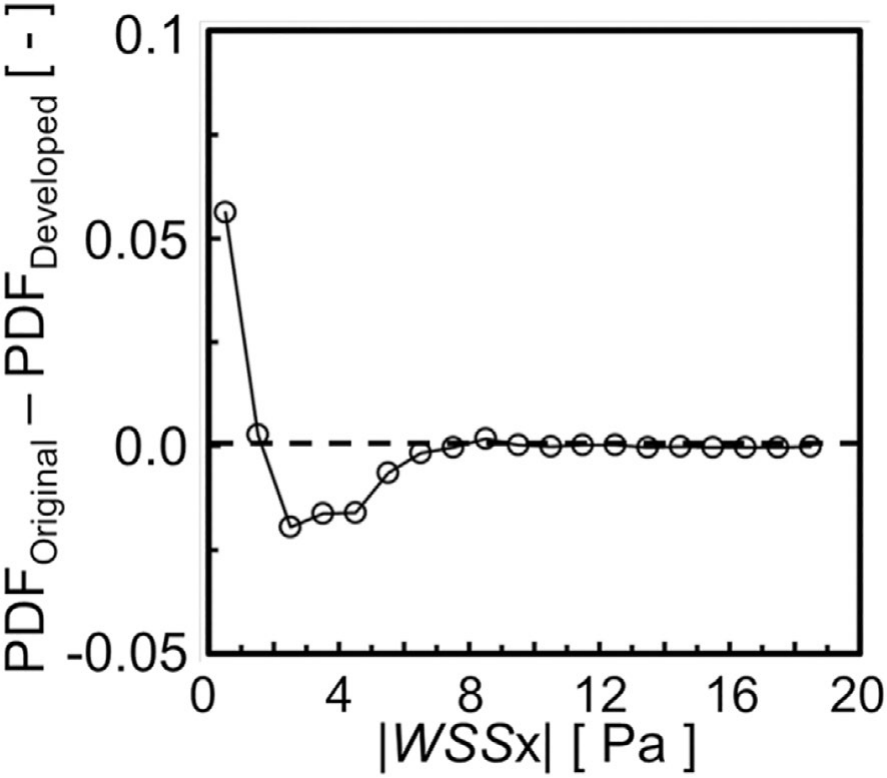
Difference in PDF of WSS in flow direction $\tau_{x}\tau_{x}$ before and after smoothing.

We investigate the effects of changes in surface roughness and volume on CFD of WSS. WSS was calculated using the following equation:

(12) $$\tau \left(x,y,z\right)=\mu \frac{u_{p}}{y_{p}}$$

where $u_{p}$ is velocity at the cell center near the wall, and $y_{p}$ is distance between the cell center and the wall surface. Figure 18 shows the WSS distribution. A comparison of the WSS distributions revealed no significant differences under laminar flow (*Re* = 444) in carotid artery. To analyze the WSS differences, the Probability Density Function (PDF) of component $\tau_{x}$ of the WSS in the flow direction was evaluated. The PDF of $\tau_{x}$ is the calculated ratio of the surface area of $\tau_{x}$ occurs in the analysis region of the entire analysis region. The sign of $\tau_{x}$ is positive in the opposite direction to the flow. Figure 19 shows the PDF of $\tau_{x}$. Figure 19a shows the PDF of $\tau_{x}$ over the entire geometry, while Figure 19b shows the PDF of $\tau_{x}$ over three quarters of the geometry. When evaluating the PDF of $\tau_{x}$ in the entire geometry, no significant difference was observed before and after smoothing. This is the entire geometry includes the area before the bifurcation where the separation vortex is not generated, and the effect of the reduced surface roughness on WSS is estimated to be minimal. Therefore, the PDF of $\tau_{x}$ is re‐evaluated by excluding the region before the bifurcation. There is a difference in the PDF of low $\tau_{x}$ when evaluated over three quarters of the geometry. Figure 20 shows the difference in the PDF of $\tau_{x}$ before and after smoothing. The probability of high $\tau_{x}$ does not show a significant difference before and after smoothing, but the probability of low $\tau_{x}$ such as −1~1 Pa is higher in the original geometry than in the smoothed geometry (Figure 20).

**FIGURE 21:**
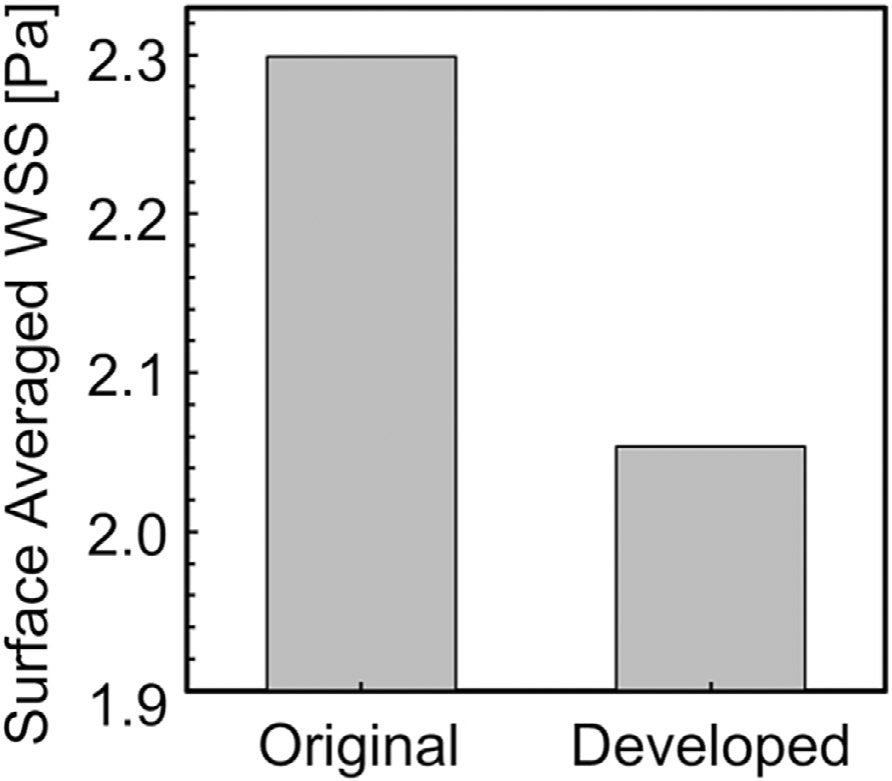
Surface‐averaged WSS before and after smoothing.

**FIGURE 22:**
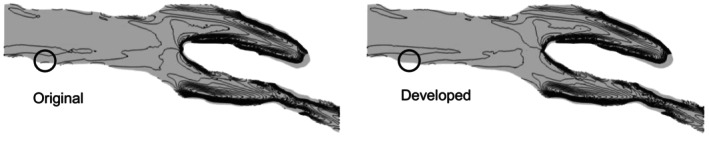
Comparison of vorticity contours in *x*‐direction before and after smoothing.

Next, to compare the WSS before and after smoothing quantitatively, the surface‐averaged WSS was evaluated. The surface‐averaged WSS is defined by the following equation:

(13) $$\bar{\mathrm{WSS}}=\frac{1}{S}\int_{S}\left|\tau \left(x,y,z\right)\right|\mathrm{dS}$$

where S is the geometric surface area. Figure 21 shows the surface‐averaged WSS for the original geometry and geometry after smoothing. It was found that the surface‐averaged WSS decreased by approximately 10.6% after smoothing (Figure 21). This is because the separation position and vortex behavior near the wall surface changed with the decrease in surface roughness. Figure 22 shows the contour lines of vorticity in the flow direction. Comparing the contour lines before and after smoothing, vortices were generated near the wall before bifurcation in the original geometry, but were not generated at the same location after smoothing. Additionally, it can be observed that the interval between contour lines is shorter in the original geometry, indicating a stronger rotational force of the vortex generated near the wall (Figure 22). These results suggest that reducing the surface roughness of the geometry suppresses the generation of small vortices in the vicinity, making it less likely to low WSS to occur. Therefore, CFD may overestimate WSS when using medical checkup images. Furthermore, since low WSS is known to cause plaque growth and formation [17, 18, 19], the results demonstrate the importance of the developed method.

## 7 Practical Verification of Developed Method

### 7.1 Evaluation of the Universality of Developed Method for Other Geometries

**FIGURE 23:**
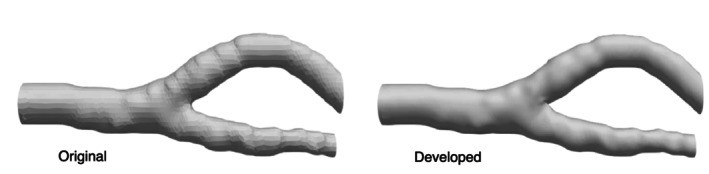
Example of geometries before and after smoothing.

**FIGURE 24:**
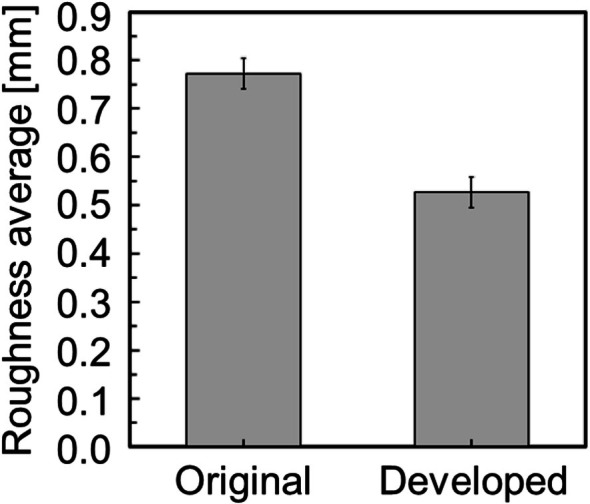
Comparison of averaged‐arithmetic mean roughness before and after smoothing at 12 cases.

**FIGURE 25:**
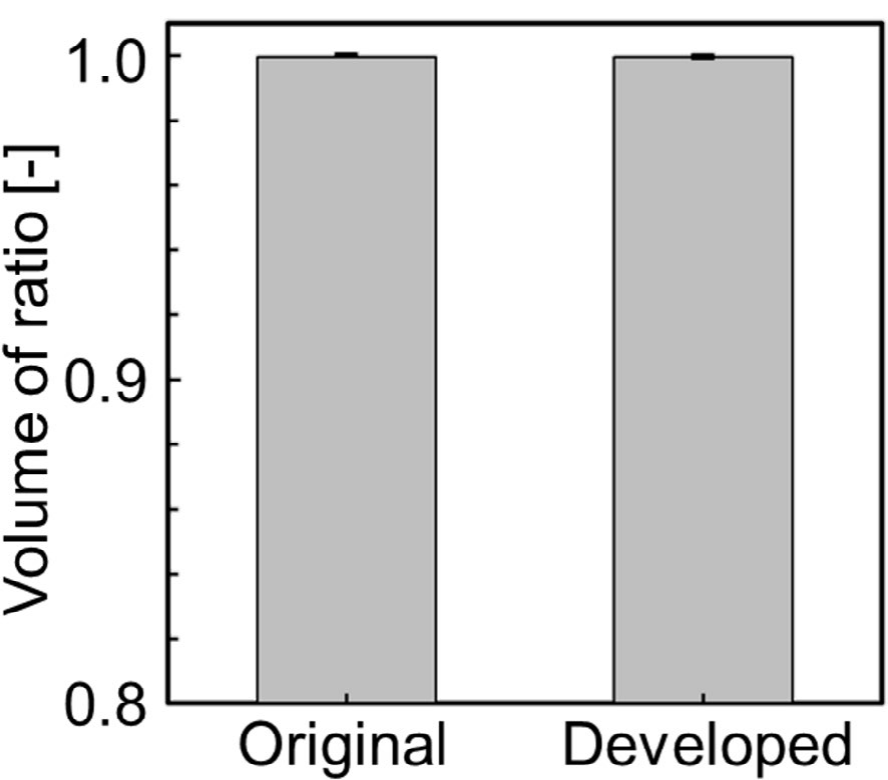
Comparison of volume ratio before and after smoothing at 12 cases.

**FIGURE 26:**
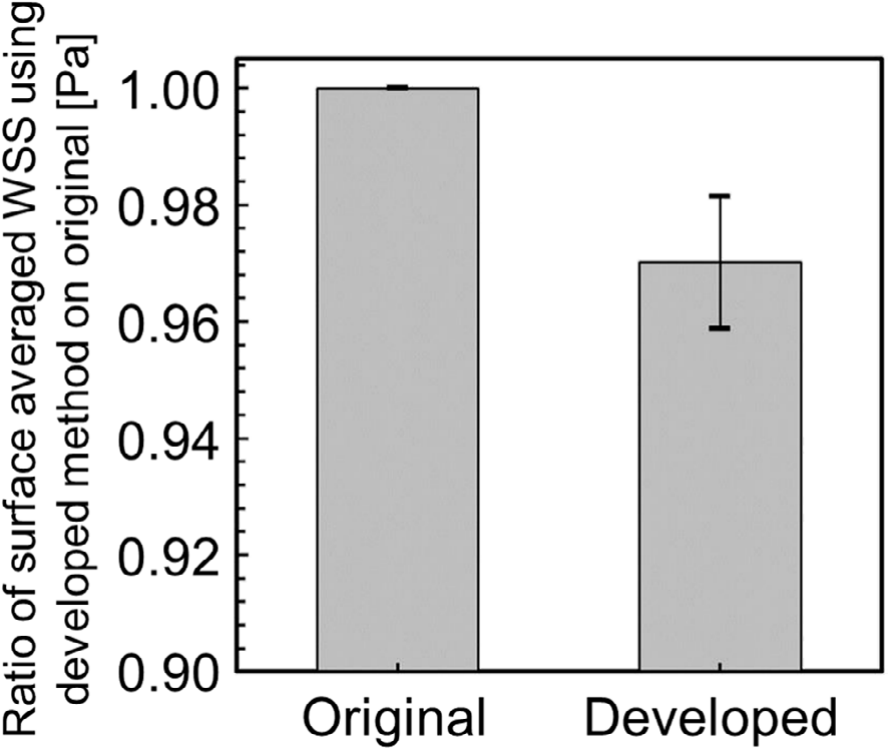
Surface‐averaged WSS before and after smoothing at 12 cases.

To evaluate the universality of the developed method for other geometries, we applied the developed method to 12 geometries of the carotid arteries and evaluated the changes in surface roughness, volume, and surface‐averaged WSS. Figure 23 shows an example of the geometry obtained by applying the developed method, while Figure 24 shows the averaged‐arithmetic mean roughness and its standard deviation for the 12 geometries. As shown in Figure 24, the averaged‐arithmetic mean roughness after smoothing can be reduced by approximately 27%–32% compared to the original geometries, even when applied to different geometries. Specifically, the averaged‐arithmetic mean roughness was 0.773 ± 0.031 mm for the original geometries and 0.528 ± 0.026 mm for the smoothed geometries. Furthermore, because the variation between the geometries was small, the developed method can produce geometries with the same arithmetic mean roughness across various cases. Figure 25 shows the volume changes for the 12 geometries along with their standard deviations. It was found that the volume change before and after smoothing was minimal, on the order of a few percent. Therefore, the developed method was shown to reduce the surface roughness of geometries obtained from medical checkup images to the same level and produce geometries with minimal volume changes. Figure 26 shows the surface‐averaged WSS for 12 geometries. Even though the surface roughness was reduced to the same level for all 12 geometries, the prediction accuracy of the surface‐averaged WSS remained at approximately 4.2% on average. This is because the averaged diameter of the 12 geometries was 7.50 mm, which is larger than the diameter shown in Figure 15 (diameter: 7.11 mm). In the case of the comparison under the same conditions of *Re*, the difference in the surface‐averaged WSS before and after smoothing decreased due to the smaller averaged velocity resulting from the larger diameter. These results confirmed the universality of the developed method. In Section 7.3, we will examine whether a similar smoothing effect can be obtained for smaller vessels using cerebral artery geometry.

### 7.2 Comparison With Conventional Method

**FIGURE 27:**
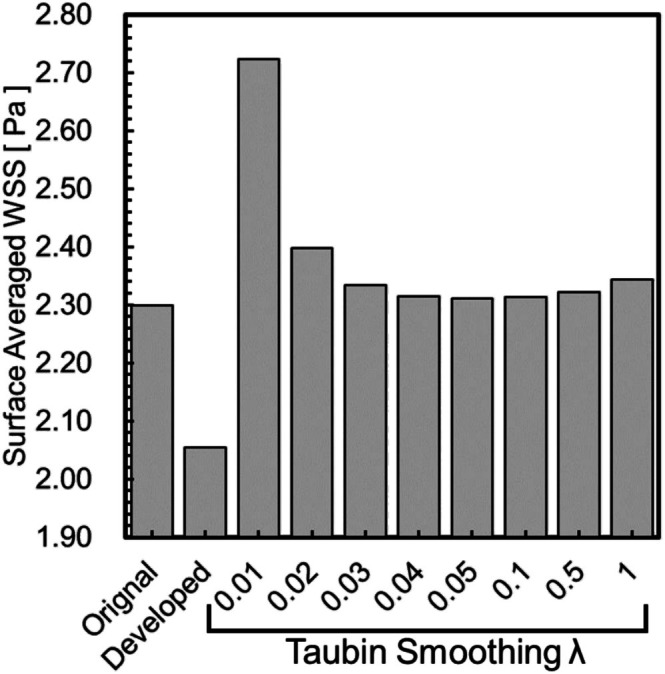
Comparison of surface‐averaged WSS with developed method and Taubin's method.

Figure 27 shows the comparison of the surface‐averaged WSS between the developed method and Taubin's method. The developed method had a smaller surface‐averaged WSS than Taubin's method under all conditions. This is because Taubin's method reduces the volume (Figure 4). The effect of the increased flow velocity by reducing the volume, which raised the WSS, was greater than the effect of reduced surface roughness, which lowered the WSS. Therefore, Taubin's method did not reduce the surface‐averaged WSS. We observed that the developed method is superior, as it achieves small surface roughness, minimal volume change, and a reduced surface‐averaged WSS.

### 7.3 Application of Developed Method to Cerebral Artery Geometry and the Evaluation

**FIGURE 28:**
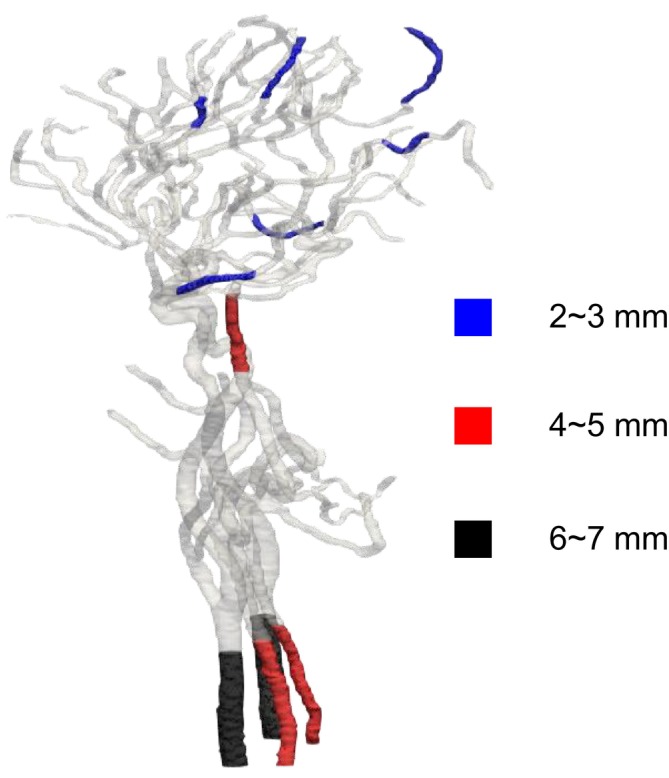
Measurement location in cerebral arteries for averaged‐arithmetic mean roughness.

**FIGURE 29:**
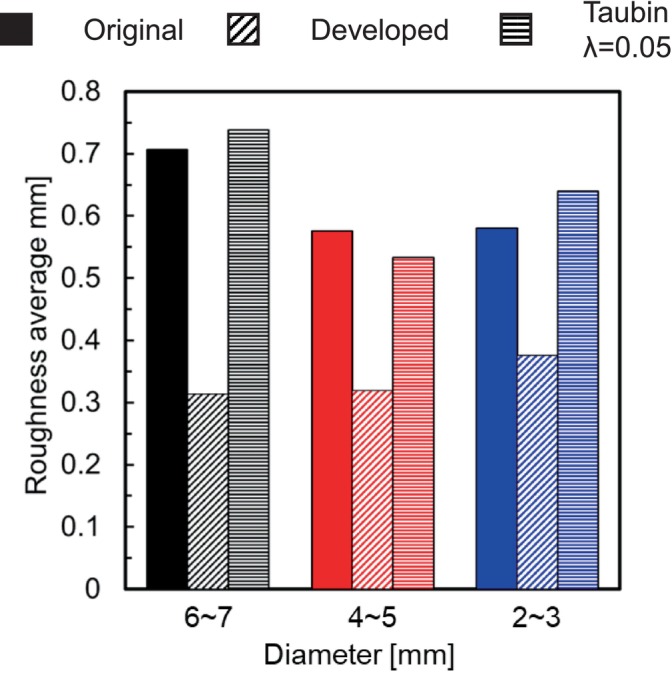
Comparison of averaged‐arithmetic mean roughness before and after smoothing in cerebral artery.

**FIGURE 30:**
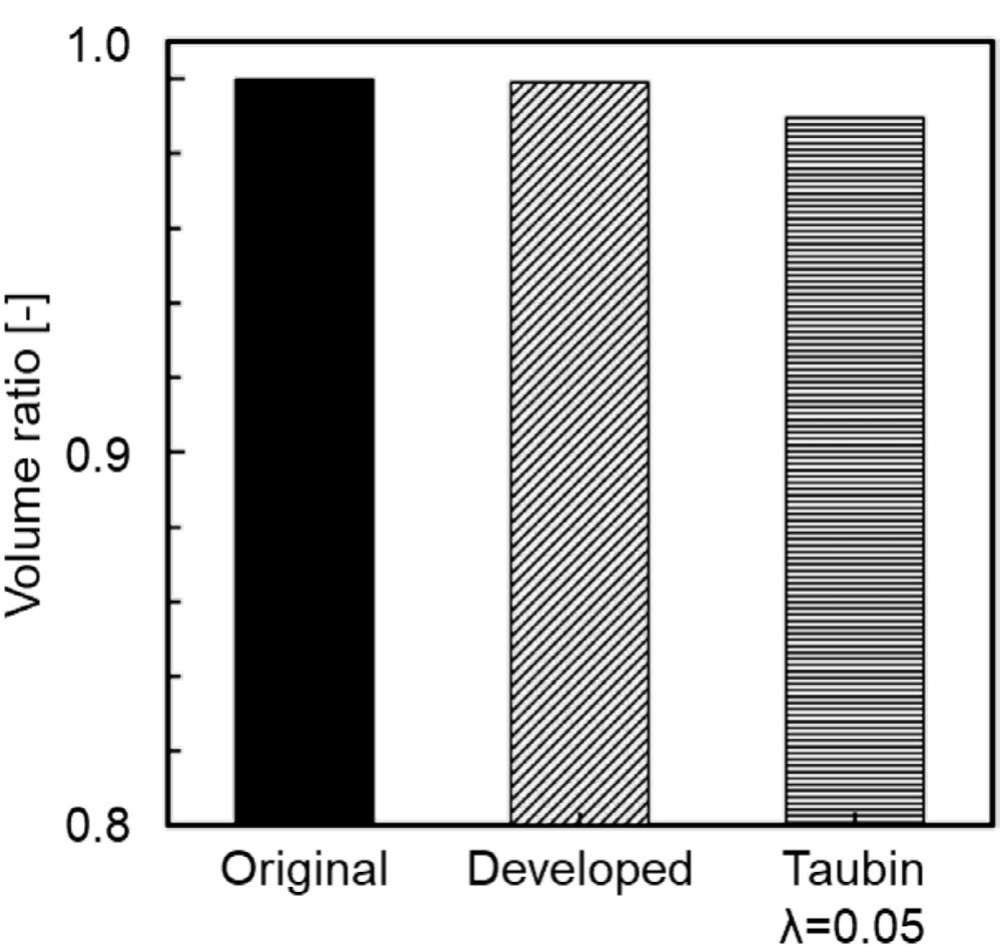
Comparison of volume ratio before and after smoothing in cerebral artery.

**FIGURE 31:**
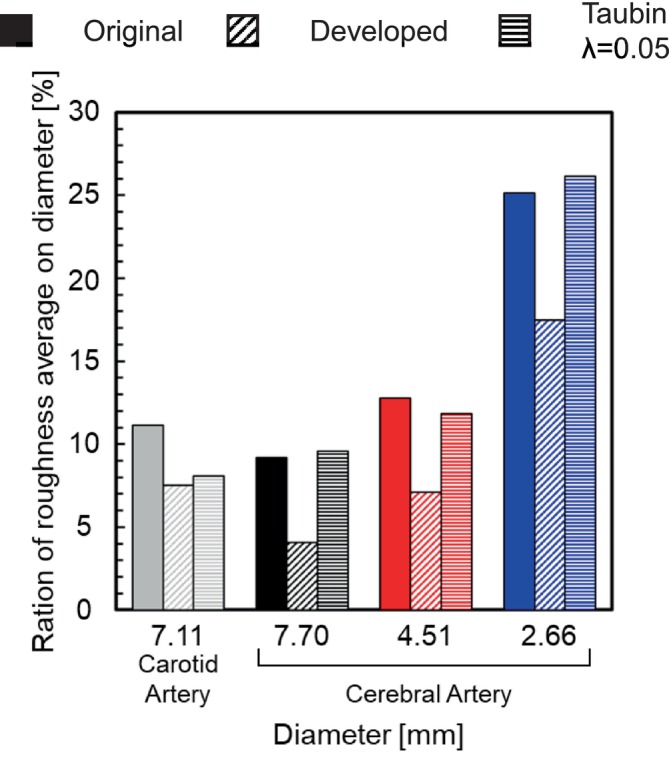
Comparison of surface roughness ratio to diameter between cerebral artery and carotid artery.

We applied the developed method to the geometry of the cerebral artery and evaluated the changes in surface roughness, volume, and WSS before and after smoothing. We also compared this method to the conventional Taubin's method. The smoothing time took approximately 25–30 min when executed on a laptop (CPU: Intel Core i5‐7200U, memory: 8 GB). As shown in Figure 27, it was found that the surface‐averaged WSS is smallest with Taubin's method when the band‐pass λ is 0.05, so the band‐pass λ was set to 0.05. Figure 28 shows the measurement position for the averaged‐arithmetic mean roughness in the cerebral artery, and Figure 29 shows the averaged‐arithmetic mean roughness. Additionally, Figure 30 shows the volume changes. The averaged‐arithmetic mean roughness values for cerebral artery diameters of 2–3 mm (averaged‐diameter: 2.64 mm), 4–5 mm (averaged‐diameter: 4.51 mm), and 6–7 mm (averaged‐diameter: 7.70 mm) were calculated using equation (7). The averaged‐arithmetic mean roughness for all cerebral artery diameters was approximately 35% smaller after smoothing, and the volume change was only a few percent. However, there is a condition with Taubin's method, where the surface roughness cannot be reduced, and the volume change reaches 3%. Next, to compare the surface roughness of the cerebral arteries with that of the carotid arteries, the ratio of surface roughness to the average diameter was calculated. Figure 31 shows a comparison of the ratio of surface roughness to the diameter of each geometry between the cerebral and carotid arteries. Compared to the carotid artery, the ratio of surface roughness to diameter decreased under all conditions when the developed method was applied.

**FIGURE 32:**
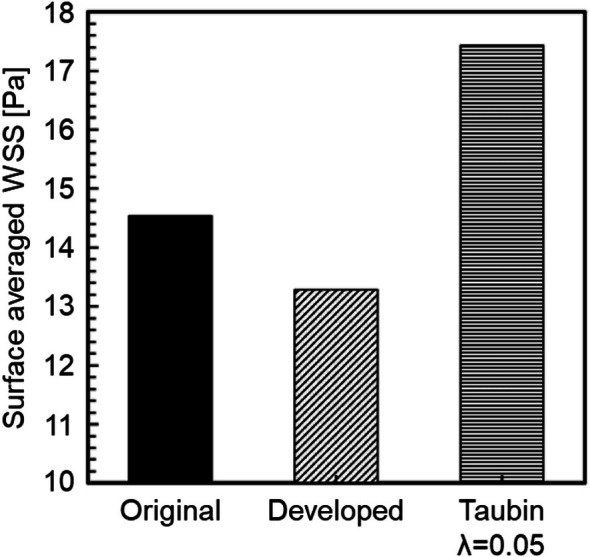
Time‐surface‐averaged WSS before and after smoothing in cerebral artery.

**FIGURE 33:**
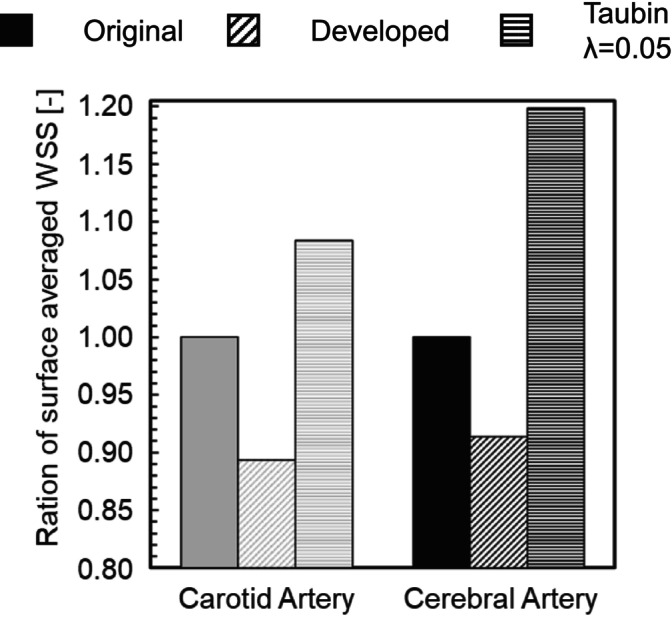
Comparison of time‐surface‐averaged WSS to diameter between cerebral artery and carotid artery.

Next, WSS was evaluated by time‐surface‐averaged WSS. Time‐surface‐averaged WSS is defined by the following equation:

(14) $$\bar{\text{TAWSS}}=\frac{1}{T}\int_{T}\bar{\mathrm{WSS}}\mathrm{dt}$$

where T is period. Figure 32 shows the time‐surface‐averaged WSS before and after smoothing. It can be observed that the time‐surface‐averaged WSS after smoothing was approximately 8.6%, the maximum time‐surface‐averaged WSS was approximately 13.5% and the minimum time‐surface‐averaged WSS was approximately 3.8% smaller than before smoothing. Conversely, the time‐surface‐averaged WSS using Taubin's method was approximately 19.9%, the maximum time‐surface‐averaged WSS was approximately 2.4% and the minimum time‐surface‐averaged WSS was approximately 8.7% larger than the original geometry. This demonstrates that the changes in surface roughness and volume have a greater effect on WSS in geometry with smaller vessel diameters. This result also indicates that the effect of smoothing influences the local distribution of WSS. Next, to compare the time‐surface‐averaged WSS in the carotid artery, the ratio of the time‐surface‐averaged WSS in the original geometry to that in the geometry after smoothing was calculated. Figure 33 shows the comparison results of the time‐surface‐averaged WSS in the carotid and cerebral arteries. The developed method was found to improve the calculation accuracy even for cerebral arteries, as it reduced surface roughness, minimized volume change, and decreased the time‐surface‐averaged WSS.

## 8 Conclusion

In this study, we developed a new method to obtain geometries with low surface roughness from low‐resolution medical checkup images by combining coordinate point interpolation with a process to selectively remove low‐frequency noise. The following conclusions were drawn:

1. The surface roughness of the geometry was reduced by approximately 27%–32% using the developed method, while the volume change remained minimal at only a few percent.
2. By applying the developed method, vortex generation near the wall was suppressed, and the WSS was reduced by approximately 4.2% compared to the original geometry.
3. A comparison with the conventional method revealed that WSS was low for the developed method.
4. It was demonstrated that this method can improve the computational accuracy of CFD for certain blood vessels, such as the carotid and cerebral arteries.

## Funding

This study was partially supported by the Co‐Funding Research Program established between Kyushu Institute of Technology and Manipal Academy of Higher Education Grant Number (Japan Society for the Promotion of Science): DST/JSPS/P‐293/2019 (JPJSBP120197726) and JST SPRING, Japan Grant Number (Japan Science and Technology Agency): JPMJSP2154.

## Ethics Statement

This study has been approved by the Institutional Ethical Committee (IEC 569–2020) of MAHE, India. The protocols of the retrospective study have been explained to the subjects who then provided the written informed consent.

## Conflicts of Interest

The authors declare no conflicts of interest.

## Data Availability

The data that support the findings of this study are available from the corresponding author upon reasonable request.
